# A Comparative Analysis of Adjunctive Transmyocardial Laser Revascularization With Coronary Artery Bypass Grafting

**DOI:** 10.7759/cureus.80968

**Published:** 2025-03-21

**Authors:** Ishan Deshmukh, Constantino G Lambroussis

**Affiliations:** 1 Vascular Surgery, Lake Erie College of Osteopathic Medicine, Elmira, USA; 2 Osteopathic Medicine/Family Medicine, Lake Erie College of Osteopathic Medicine, Elmira, USA

**Keywords:** adult cardiac surgery, coronary artery bypass grafting (cabg), coronary vessel disease, multivessel coronary artery disease (mvcad), transmyocardial revascularization (tmr)

## Abstract

Coronary artery disease (CAD), a chronic condition, is a leading cause of morbidity and mortality worldwide. It is characterized by plaque buildup in the coronary arteries, which can restrict blood flow and result in symptoms that can include chest pain and shortness of breath. Management of CAD often involves a combination of lifestyle modifications, medications, and revascularization such as coronary artery bypass grafting (CABG), percutaneous coronary intervention (PCI), and transmyocardial laser revascularization (TMLR). This meta-analysis examines the short-term and long-term benefits and outcomes of TMLR on angina severity when used as an adjunct to CABG in patients with CAD. Following the guidelines of the Preferred Reporting Items for Systematic Reviews and Meta-Analyses, a comprehensive literature search and analysis was performed in the PubMed, BioMed Central, and ClinicalTrials.gov databases for relevant studies from database inception to February 2023. A comprehensive literature review and meta-analysis of current peer-reviewed studies suggest that adjunctive TMLR + CABG can significantly reduce angina severity and improve clinical outcomes for patients with CAD in the short term. Benefits include decreased lengths of stay in the intensive care unit, hospital length of stay, decreased operative mortality, and superior angina relief compared to CABG alone. Certain studies that evaluated long-term outcomes indicated the benefits of adjunctive CABG and TMLR were lost after one year; however, further investigation regarding long-term outcomes would be beneficial.

## Introduction and background

Angina pectoris, a symptomatic manifestation of coronary artery disease (CAD), poses a multifaceted challenge to cardiovascular health experts. Characterized by chest pain or discomfort, angina signals inadequate blood flow to the heart muscle, necessitating a nuanced understanding of its underlying pathophysiology. In the realm of interventions, coronary artery bypass grafting (CABG) emerges as a cornerstone for experts addressing compromised coronary circulation. This intricate surgical procedure aims to not only restore blood flow but also alleviate anginal symptoms, improve cardiac function, and enhance patients’ overall quality of life.

Typically, in cases of CAD, pharmacotherapy is preferred as the first-line therapeutic line of medical management, and revascularization attempts, such as percutaneous coronary intervention (PCI) and CABG, are generally indicated in refractory cases. PCI involves cardiac catheterization and increasing the diameter of stenotic coronary arteries using an inflatable balloon [1]. However, despite the widespread utilization of these conventional revascularization techniques, a subset of patients continues to experience debilitating angina symptoms, defined as refractory angina.

CABG is a well-established surgical procedure used to treat patients with severe CAD. CABG is indicated under Class 1 recommendations of the 2011 American College of Cardiology Foundation/American Heart Association guidelines for left main vessel stenosis greater than 50%, three-vessel CAD involving 70% stenosis with or without the involvement of the proximal segment of the left anterior descending artery (LAD), two-vessel disease involving the LAD and a secondary vessel, single or multivessel stenosis with more than 70% occlusion with anginal symptoms refractory to maximal medical therapy, and single-vessel stenosis with more than 70% occlusion in a patient who survived at least one instance of cardiac arrest from ischemia related ventricular tachycardia [2]. The typical CABG procedure involves harvesting conduit vessels from various sources, including the left internal thoracic/mammary artery, the right internal thoracic/mammary artery, the radial artery, the saphenous vein, and the right gastroepiploic artery. These conduit vessels are then used to create artificial bypass routes distal to areas of stenosis. During the procedure, the heart is placed into arrest using high-potassium cardioplegic solutions while the patient simultaneously undergoes cardiopulmonary bypass [3]. The CABG procedure is associated with a wide range of potential complications, both intraoperative and postoperative. These complications can include atrial fibrillation, which occurs in up to 24% of CABG patients, as well as pleural effusion, mediastinitis, sternal osteomyelitis, graft failure, renal dysfunction, stroke, bleeding events, myocardial dysfunction or ischemia, and multisystem organ dysfunction due to the systemic inflammatory response triggered by the effects of cardiopulmonary bypass [4]. Given the significant morbidity associated with CABG, it becomes imperative to explore alternative revascularization strategies that can provide effective relief of angina symptoms while minimizing the risks posed by conventional approaches.

Despite the effectiveness of conventional revascularization techniques such as CABG and PCI in managing CAD and alleviating angina, a subset of patients continues to experience refractory angina, defined as persistent disabling chest pain despite optimal medical therapy and revascularization [1]. Recognizing the limitations of conventional CABG, such as its invasive nature and associated morbidity, the medical community has sought alternative and adjunctive surgical approaches that can minimize invasiveness while preserving the benefits of myocardial revascularization.

One such innovative procedure is transmyocardial laser revascularization (TMLR), which was performed in 1995 [5]. TMLR involves creating a series of small channels through the myocardium using laser energy, thereby improving blood flow to the ischemic myocardium and reducing anginal symptoms in patients with refractory angina who are not candidates for conventional revascularization. The premise underlying TMLR is that the laser-created channels can serve as conduits, allowing oxygenated blood from the left ventricular cavity to directly penetrate and perfuse the ischemic regions of the myocardium, thereby alleviating the underlying cause of angina [6-8]. The TMLR procedure is designed to improve blood flow to ischemic regions of the myocardium. During the procedure, the surgeon creates a series of small, 1-2 mm diameter channels through the left ventricular free wall and into the myocardium using a carbon dioxide or holmium:YAG laser [9]. These laser-created channels span from the ventricular cavity directly to the ischemic areas of the heart muscle, allowing oxygenated blood to directly perfuse the previously compromised tissue [8]. The primary intended outcome of TMLR is to reduce the severity of angina symptoms in patients with refractory CAD who are not suitable candidates for traditional revascularization techniques, such as CABG or PCI [10]. By enhancing myocardial perfusion through the creation of these laser channels, TMLR aims to alleviate the underlying cause of angina and improve the overall quality of life for these patients.

Although the exact mechanisms by which TMLR improves myocardial perfusion and anginal symptoms are not fully elucidated, several proposed theories have emerged from the available evidence. The traditional hypothesis was that the laser-drilled channels would remain patent and serve as direct conduits for blood flow from the ventricular cavity into the ischemic myocardium. However, subsequent studies have challenged this notion, demonstrating that the channels tend to close and become occluded within a relatively short period [11]. More recent investigations suggest that the primary mechanism of action of TMLR may be related to the induction of angiogenesis, or the growth of new blood vessels, within the myocardium. The trauma and tissue disruption caused by the laser channels may stimulate the release of various growth factors, leading to the formation of new capillaries and improved regional myocardial perfusion. Other proposed mechanisms include a degree of denervation caused by the laser injury, which may reduce the perception of anginal pain, as well as a potential placebo effect observed in some patients undergoing this procedure [11,12]. Ultimately, despite the ongoing debate surrounding the precise mechanisms of action, the available evidence suggests that TMLR has emerged as a promising therapeutic option for patients with refractory angina who are not candidates for conventional revascularization.

There are several complications of the TMLR procedure, including cardiac arrhythmia, bleeding events, myocardial perforation, and pericardial effusion. However, when performed by experienced surgeons, the procedure is generally well-tolerated, with low rates of mortality and morbidity compared to conventional CABG. Over the past two decades, TMLR has been the subject of extensive research and clinical investigation, with more recent studies showing perioperative mortality at 1% to 7% with major adverse cardiac event (MACE)-free rates at 15 years post-procedure at 63% [13,14].

While many of the methods of revascularization have their benefits, adjunctive surgical techniques present an intriguing alternative approach for patients with severe, refractory angina. In particular, adjunctive CABG and TMLR procedures have demonstrated the potential to alleviate angina symptoms and improve outcomes in patients who are not candidates for conventional revascularization. Prior meta-analyses have utilized only two studies that looked at the adjunctive use of TMLR with CABG, which found no statistically significant difference in mortality rates between the patients who underwent the combined TMLR-CABG procedure and those who received CABG alone, but did report a significantly greater reduction in angina severity among the patients who received the combined TMLR-CABG intervention [15]. This systematic review serves to provide an updated comprehensive evaluation of the current evidence surrounding the use of TMLR as an adjunct to CABG in the management of refractory angina.

## Review

Methodology

**Figure 1:**
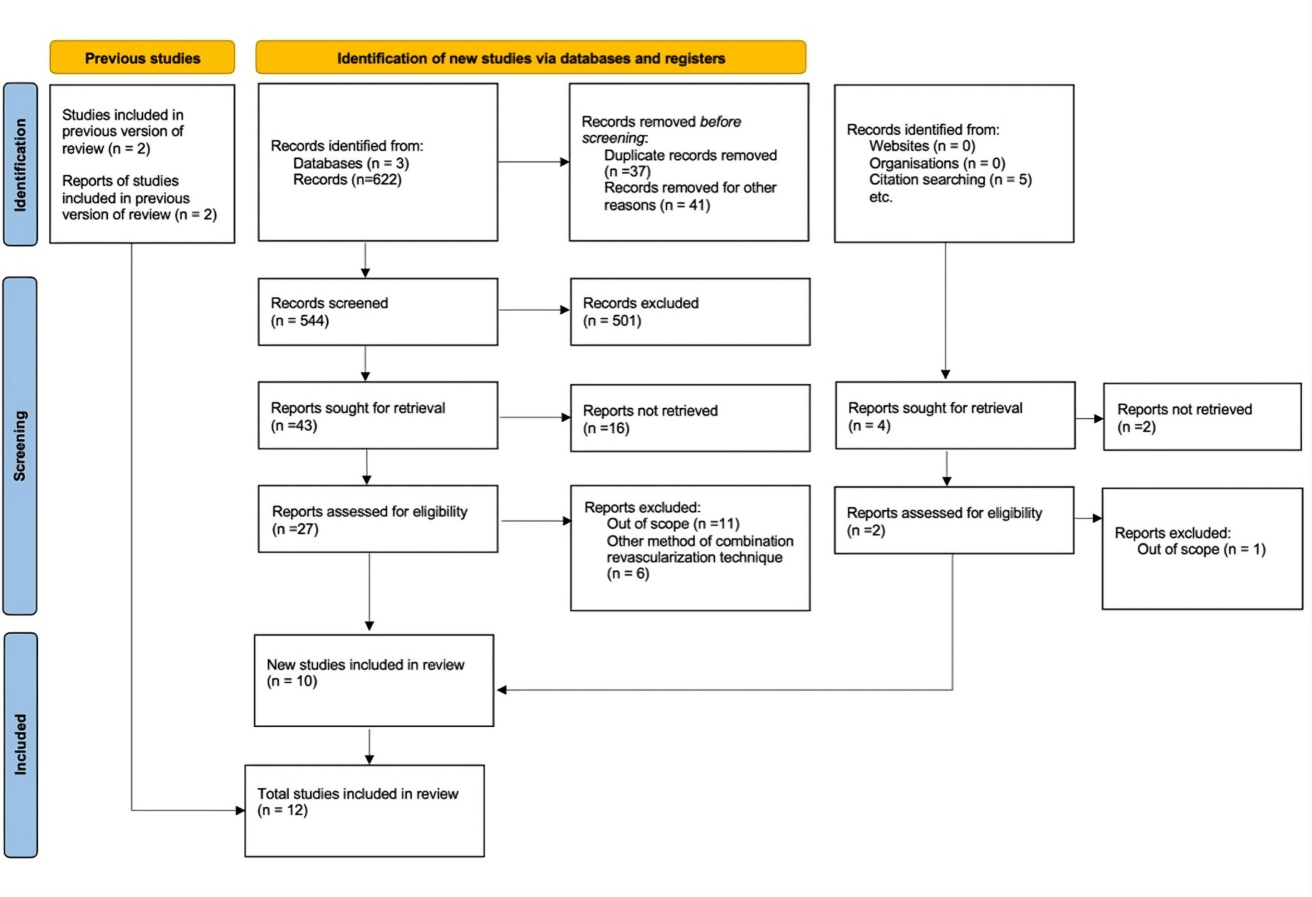
Preferred Reporting Items for Systematic Reviews and Meta-Analyses (PRISMA) flowchart for literature search and selection process.

This systematic review and meta-analysis was conducted in accordance with the Preferred Reporting Items for Systematic Reviews and Meta-Analyses (PRISMA) guidelines [16]. A comprehensive search of the PubMed, BioMed Central, and Clinicaltrials.gov databases was performed for relevant studies from database inception to February 2023 (Figure 1). The keywords used to identify potential records included “transmyocardial laser revascularization,” “TMR,” “TMLR,” “coronary artery bypass graft,” “refractory angina,” “revascularization techniques,” and “adjunctive revascularization techniques.” The primary objective of this meta-analysis was to evaluate the efficacy and investigate the reduction of angina severity of TMLR as an adjunct to CABG compared to CABG alone in the management of patients with refractory angina. The secondary objectives were to assess the impact of the combined procedure on mortality, MACE, and quality of life outcomes. Inclusion criteria for the study were randomized controlled trials, cohort studies, and case-control studies that evaluated the safety and efficacy of combining TMLR and CABG compared to CABG alone in patients with refractory angina. Eligible studies reported on clinical outcomes, such as mortality, myocardial infarction, repeat revascularization, and angina severity, and were available in the English language. Exclusion criteria encompassed studies that did not directly compare the combined TMLR and CABG procedure to CABG alone, as well as those involving patients with acute coronary syndromes or other significant comorbidities. Review articles, editorials, and case reports were also excluded.

Evidence of the safety and efficacy of conjunctive TMLR and CABG

Trehan et al. reported a case series that examined the reduction in angina symptoms and the overall safety and efficacy of combining TMLR and CABG in patients with diffuse CAD [17]. In their study, 56 patients, with an age range from 37 to 81 years, a mean age of 56, and 50% of patients having an angina severity class of 3 and 4, underwent the combined TMLR and CABG procedure without cardiopulmonary bypass. The internal mammary artery (IMA) was used as the primary conduit in all patients, and TMLR was performed intraoperatively using a 1,000 W carbon dioxide laser on areas of the myocardium not amenable to surgical bypass. One patient died due to intractable ventricular arrhythmia intraoperatively leading to a mortality rate of 1.8%. At the one-year follow-up, 90.9% of patients were free from angina, and myocardial perfusion scans revealed a significant improvement from a preoperative average of 52% to 91%. Furthermore, the patients’ average functional capacity, as measured by the Karnofsky score, increased substantially from a baseline of 44% to 86% at one year post-procedure. Likewise, their average metabolic equivalent improved from 4.5 at baseline to 9.4 at the one-year mark. Trehan et al. again published a second study evaluating the safety, efficacy, and impact of combining TMLR with CABG on anginal symptoms and myocardial perfusion in 77 patients [18]. In this patient group, 84% of patients underwent a mid-sternotomy approach and 16% underwent a left anterior thoracotomy, with all patients receiving TMLR intraoperatively on areas of the myocardium not amenable to surgical revascularization. One (1.3%) patient died postoperatively due to ventricular arrhythmia. The study reported an overall improvement in anginal symptoms, with 89% of patients being free from angina at the one-year follow-up. Metabolic stress testing revealed an improvement in exercise capacity, with the average time increasing from 5.2 minutes at baseline to 9.7 minutes one year later. Myocardial perfusion scanning also demonstrated a significant and steady improvement in perfusion, with a 28.4% gain in areas where TMLR was performed, as measured every three months postoperatively in the grafted segments.

The studies listed in Table 1 demonstrate the feasibility, safety, and efficacy of incorporating TMLR as an adjunct to CABG procedures in patients with severe, diffuse CAD and refractory angina not amenable to conventional revascularization.

**Table 1:** Studies evaluating the short-term outcomes of conjunctive TMLR and CABG. The table presents an overview of the studies that assessed the comparative short-term results and outcomes of conjunctive TMLR and CABG regarding operative feasibility, safety, and a one-year analysis of cardiac and metabolic function. Both studies authored by Trehan et al. indicated a significant reduction of angina severity and exercise tolerance and increases in cardiac perfusion with a slight mortality risk due to ventricular arrhythmia. MET = metabolic equivalents of task; KPS = Karnofsky Performance Score; CABG = coronary artery bypass grafting; TMLR = transmyocardial laser revascularization

Authors	Sample size	Method of study	Follow-up time	Method of assessment	Findings
Trehan et al. (1997) [17]	N = 56	TMLR + CABG in all patients with results compared to baseline	12 months	Operative mortality, angina severity, myocardial perfusion scanning, metabolic stress test, MET, KPS	Operative mortality of 1.8%. 90.9% of patients were angina-free by 12 months. Improvement of reversible ischemia from 52% to 91% at 12 months. Exercise tolerance increased from 5.2 minutes to 9.4 minutes at 12 months. MET increased from 4.5 to 9.4 at 12 months. KPS increased from 44% to 86% at 12 months
Trehan et al. (1998) [18]	N = 77	TMLR + CABG in all patients with results compared to baseline	12 months	Operative mortality, angina severity, metabolic stress test, myocardial perfusion scanning	Mortality of 1.3%. 89% of patients were angina-free by 12 months. Increased exercise tolerance from 5.2 minutes to 9.7 minutes at 12 months. 28.4% showed improvement in reversible ischemia

Trials assessing the benefits of adjunctive TMLR-CABG compared to CABG alone

Prior meta-analyses have examined the outcomes of combining TMLR and CABG in patients with severe, diffuse CAD but have included only two studies that directly compared the combined procedure to CABG alone. In 2000, Allen et al. published the results of a randomized controlled trial comparing TMLR plus CABG (N = 132) to CABG alone (N = 131) in 263 patients [19]. In the combined therapy arm, the intraoperative mortality was 1.5% compared to 7.6% in the CABG-only group. Furthermore, patients in the combined therapy arm were reported to require less postoperative inotropic support as well as a lower rate of intra-aortic balloon pump utilization. At the one-year follow-up, the authors reported no significant differences in the reduction of angina severity or improvement in exercise tolerance between the combined TMLR and CABG group and the CABG-alone group. However, patients who underwent the combined TMLR and CABG procedure had a better cumulative MACE-free incidence rate and overall lower mortality rate, as demonstrated by Kaplan-Meier analysis.

In a five-year follow-up study, Allen et al. reported that 83% of the original cohort was available for analysis, conducted across 13 surgical centers [20]. The combined TMLR and CABG group demonstrated superior angina relief, with no patients experiencing class 3 or 4 angina compared to 10% in the CABG-alone group (p = 0.009). Additionally, the combined therapy group exhibited a lower overall angina score and a greater proportion of patients achieving an angina-free state. Specifically, the authors noted that the combined TMLR and CABG group achieved significantly better outcomes in terms of angina severity, with no patients reporting class 3 or 4 angina compared to 10% in the CABG-alone group. Furthermore, the combined therapy group had a lower overall angina score and a higher proportion of patients becoming completely free of anginal symptoms. The overall findings from this long-term study suggest that the addition of TMLR to CABG provides superior angina relief in addition to an increased early mortality benefit compared to CABG alone in patients with diffuse CAD and incomplete revascularization [21].

In 2004, Frazier et al. published the results of a similar randomized controlled trial that compared TMLR + CABG (n = 23) to CABG alone (n = 21) in 44 patients across four surgical centers [22]. The intraoperative CABG procedure was similar in both procedural arms with patients in the TMLR + CABG group receiving an additional 25 + 11 laser channels in areas of the myocardium not amenable to surgical bypass. The 30-day mortality rate following the surgical procedure was reported to be 13% in the combined TMLR and CABG therapy group compared to a higher rate of 28% in the CABG-alone group. The patients in both study arms were followed for one year and four years after the initial procedure. While both groups demonstrated improvements in angina severity and exercise tolerance, the authors found no statistically significant difference in the overall mortality rates between the two groups at the four-year follow-up. However, there was a notable difference in the rates of repeat revascularization procedures, with the TMLR + CABG group requiring zero additional revascularization procedures compared to the CABG-alone group (24%) over the long-term follow-up period. In summary, the studies evaluating the combination of TMLR and CABG suggest that this combined approach may confer meaningful advantages in certain high-risk patient populations with diffuse CAD and incomplete revascularization and may lead to a decreased risk of requiring further coronary intervention.

In 2015, Berishvili et al. published the results of a larger study evaluating the results of 831 patients who needed cardiac revascularization due to CAD [23]. The study compared three surgical cohorts, i.e., 711 patients with CAD but without diffuse coronary lesions who underwent CABG alone, 33 patients with advanced CAD with diffuse lesions who underwent CABG alone, and 87 patients with advanced CAD and diffuse lesions who underwent the combined TMLR and CABG procedure. The researchers found that patients with advanced CAD and diffuse lesions who underwent CABG alone had a higher rate of critical coronary arterial spasm, with one-third of this group experiencing this adverse effect. In contrast, the combined TMLR and CABG therapy cohort had a significantly lower rate of critical coronary arterial spasms, at only 1.15%. This suggests that the addition of TMLR to CABG may help mitigate the risk of critical coronary artery spasms in patients with advanced, diffuse CAD. Additionally, patients in the combined therapy group had the lowest mortality at 1.15% compared to 12% in the CABG-alone group with advanced disease and 2% in the CABG-alone group without diffuse disease. The findings of the Berishvili et al. study suggest that the combined use of TMLR and CABG may offer meaningful clinical benefits for select high-risk patients with severe, diffuse CAD and incomplete revascularization. Specifically, this combined approach appears to be associated with improved mortality rates, a decreased risk of critical coronary arterial spasms, and a lower rate of adverse cardiovascular events compared to CABG alone in this patient population. The study findings indicate that the addition of TMLR to CABG may help mitigate the increased risks faced by patients with advanced, diffuse CAD, potentially offering an important treatment option for this complex patient cohort.

Loubani et al. published the results of a smaller randomized controlled trial in 2003 that compared the combined procedure of TMLR and CABG to CABG alone in 20 patients from a single surgical center. In this study, 10 patients were randomly assigned to undergo the combined TMLR and CABG procedure, while the other 10 underwent CABG alone [24]. Procedural success was measured by treadmill exercise stress testing performed at 6, 18, and 36 months post-procedure, as well as dobutamine stress echocardiography, with left ventricular myocardial wall motion score index measurements evaluated at 18 months post-procedure. The authors reported that the TMLR + CABG group exhibited significant and sustained improvements in exercise treadmill testing compared to the CABG-alone control group up to the 18-month follow-up. However, this benefit was not maintained at the 36-month evaluation. The other parameters used to assess myocardial perfusion and contractility (dobutamine stress echocardiography) did not reveal any significant differences between the two procedural arms.

Wehberg et al. published the results of a retrospective analysis in 2003 of a cohort of 255 patients undergoing cardiac revascularization with either CABG alone (n = 219) or conjunctive CABG plus TMLR (n = 36) [25]. The authors reported that there were no mortalities in the CABG + TMLR group but the rates of other major adverse outcomes, including bleeding events, respiratory distress, acute renal failure, and stroke, were similar between the two procedural groups. They did find, however, that the CABG + TMLR group had a significantly shorter intensive care unit and hospital length of stay compared to the CABG-alone group. Furthermore, the CABG + TMLR cohort had a significantly lower 30-day readmission rate of 2.8% compared to 7.8% in the CABG-only group. Similarly, the CABG + TMLR group experienced a significantly lower frequency of atrial fibrillation. Overall, the retrospective analysis by Wehberg et al. suggested that the addition of TMLR to CABG may confer short-term procedural benefits in terms of postoperative outcomes and healthcare utilization without adversely impacting longer-term survival or freedom from cardiovascular events. While the addition of TMLR to CABG appears to provide some potential benefits in terms of angina relief, repeat revascularization, and short-term postoperative outcomes, the long-term survival impact remains uncertain based on the conclusions of the studies mentioned so far.

In a 2003 study, Dziuk et al. evaluated the effects of combining CABG with TMLR compared to TMLR alone [26]. The researchers assessed various outcomes, including angina severity using the Canadian Cardiovascular Society grading system, exercise tolerance measured by radionuclide ventriculography, and left ventricular ejection fraction. Measurements were taken before the procedures and at a six-month follow-up after the interventions. Overall, 62 patients were included in the study with 38 patients undergoing the TMLR procedure and 24 receiving the combined TMLR and CABG intervention. The authors reported that both treatment groups experienced significant improvements in angina severity and exercise tolerance but were non-different from each other at the six-month follow-up. However, the left ventricular ejection fraction demonstrated a significant decrease in both groups with a mean of 61% pre-intervention to 54% at six months post-intervention. Overall, this study suggests that while the combined TMLR and CABG approach may be safe and feasible, it does not appear to confer any additive benefits over TMLR alone in the short term.

In 2000, Lutter et al. published the results from a study comparing TMLR alone (n = 45), CABG alone (n = 35), and a combination of TMLR and CABG (n = 17) in patients with CAD and severe angina to evaluate differences in treadmill stress testing, echocardiography, ventriculography, radionuclide imaging with assessment of ventricular perfusion and metabolism, and hemodynamic assessment [27]. The findings demonstrated significant improvements in the severity of angina by the Canadian Cardiovascular Society class and New York Heart Association functional assessment, as well as improved exercise capacity for all three treatment groups at the six- and 12-month follow-up periods; however, the other parameters were unchanged in all cohorts. The authors also reported that only the CABG cohort appeared to have an improvement in stress perfusion studies, which led them to conclude that the combination of TMLR and CABG improved parameters of treadmill stress tolerance, exercise capability, and sustained improved clinical outcomes but had little effect on the other measures of ventricular function and myocardial perfusion in this very high-risk patient population.

Long-term results

In 2018, Konstanty-Kalandyk et al. published the results of a retrospective study that followed 46 patients who received conjunctive TMLR + CABG therapy on at least one heart wall compared to a matched historical single therapy group of 40 patients who received either TMLR on at least a single heart wall or CABG [28]. The authors further stratified the results of the patients who received TMLR in both the combined therapy and single therapy groups and identified survival benefits on whether the TMLR procedure was performed on the anterior, lateral, or posterior heart wall. The main finding of the study was that patients who underwent TMLR on the anterior heart wall in addition to CABG exhibited a significantly improved 10-year survival rate of 100% compared to only 72% in the TMLR-alone or CABG-alone groups. Kaplan-Meier analysis demonstrated a clear survival benefit with the combined TMLR and CABG procedure when performed on the anterior heart wall. This suggests that the location and distribution of TMLR channels performed may be an important determinant of the long-term survival benefit provided by the combined TMLR and CABG approach.

Quality of life

The quality of life and morbidity of patients who receive combined TMLR and CABG compared to CABG alone has also been previously evaluated by Guleserian et al. in 2003 [29]. The researchers assessed quality of life and angina scores using the Seattle Angina Questionnaire in addition to age-matched comparison in a cohort of 20 patients who underwent CABG alone. In this study, 81 patients were evaluated over 24 months, with 34 patients receiving sole TMLR therapy and 47 receiving the combined TMLR and CABG procedure. The patients were further stratified into high-risk cohorts of left ventricular dysfunction with an ejection fraction of less than or equal to 40% (n = 37), unstable angina (n = 30), or congestive heart failure (n = 33). The study demonstrated that patients in the combined TMLR and CABG group had significantly greater improvements in quality of life and angina scores relative to the TMLR-only group. Furthermore, the 18-month survival rate of patients in the stratified high-risk subgroups was decreased in the cohort of patients who had left ventricular wall dysfunction or congestive heart failure. In summary, the authors found an increased quality of life and relief of anginal symptoms for patients who received the combined TMLR and CABG procedure compared to TMLR alone, but caution should be taken when considering this approach in patients with left ventricular dysfunction or congestive heart failure. Table 2 presents an overview of the studies incorporated in the systematic review with relevant and pertinent findings.

**Table 2:** A comparative review of conjunctive TMLR and CABG versus monotherapy revascularization. The table provides an overview of all clinical trials and studies that review the outcomes and results of conjunctive TMLR and CABG to a single surgical monotherapy revascularization, primarily CABG alone. Ten trials were utilized with a study period ranging from perioperatively to 10-year post-revascularization procedure. These studies utilized a variety of methods of assessment, including operative morbidity and mortality, the need for intraoperative cardiac supportive measures, post-procedure mortality as a measure of Kaplan-Meier analysis and survival rates by cohort, angina score, freedom from MACE, exercise tolerance, myocardial perfusion, rates of readmission and repeat revascularization procedures, length of stay in the hospital and intensive care unit, and ventricular wall motion and function. MACE = major adverse cardiac event; CHF = congestive heart failure; LVEF = left ventricular ejection fraction; CABG = coronary artery bypass grafting; TMLR = transmyocardial laser revascularization; ICU = intensive care unit; AFIB = atrial fibrillation; NYHA = New York Heart Association

Authors	Sample size	Follow-up time	Method of assessment	Findings
Allen et al. (2000) [19]	263 patients total: TMLR + CABG (n =132) and CABG alone (n = 131)	12 months	Operative mortality, postoperative inotropic support, Kaplan-Meier survival analysis, freedom from MACE, angina severity, exercise treadmill scores	Decreased operative mortality in TMLR + CABG (1.5%) vs. CABG (7.6%). Less inotropic support was used in TMLR + CABG (30%) vs. CABG (55%), with a trend of fewer intra-aortic balloon pumps used in TMLR + CABG. 1 year Kaplan-Meier analysis revealed a 95% survival rate with TMLR + CABG vs. 89% CABG only. TMLR + CABG rate of freedom from MACE 92% vs. 86% CABG. Angina and exercise scores were similar after 12 months for both groups
Allen et al. (2004) [20]	218 patients: TMLR + CABG (n = 110) and CABG alone (n = 108)	5 years	Angina score, Kaplan-Meier survival analysis	TMLR + CABG had lower mean angina score: (0.4) vs. CABG (0.7), a lower proportion of patients with class 3/4 angina (0%) vs. CABG (10%), angina-free patients (78%) vs. CABG (63%). Kaplan Meier analysis TMLR + CABG (76%) vs. CABG (80%)
Frazier et al. (2004) [22]	44 patients: TMLR + CABG (n = 23) and CABG only (n = 21)	4 years	Mortality, angina severity reduction, rates of repeat revascularization	TMLR + CABG <30-day mortality rate 13% vs. 28% in CABG. No significant difference in four-year mortality between groups. Four-year repeat revascularization 0% TMLR + CABG vs. 24% in CABG. Overall four-year adverse event-free survival rate 39% TMLR + CABG vs. 14% CABG
Berishvili et al. (2015) [23]	831 patients. Group 1: CABG without diffuse coronary artery lesions (n = 711). Group 2: CABG with diffuse coronary vessels (n = 33). Group 3: TMLR + CABG (n = 87)	N/A	Intraoperative and postoperative mortality, MACE rates	Operative mortality rate: 2.8% Group 1, 12.1% Group 2, 1.15% Group 3. Incidence of critical coronary vasospasm: 1.7% Group 1, 33.3% Group 2, 1.15% Group 3. Incidence of myocardial infarction: 0.6% Group 1, 20.3% Group 2, 1.15% Group 3
Loubani et al. (2003) [24]	20 patients: CABG only (n = 10) and TMLR + CABG (n = 10)	36 months	Angina score, exercise tolerance, wall motion score index	Similar angina scoring. Exercise increased by 199.2 seconds TMLR + CABG vs. 46.8 seconds CABG. No significant difference with stress wall motion testing
Wehberg et al. (2003) [25]	255 patients: TMLR + CABG (n = 36) and CABG alone (n = 219)	30 days	30-day outcomes, ICU length of stay, hospital length of stay, 30-day readmission rate, reduction of MACE	ICU stay of 1.6 days TMLR + CABG vs. 2.1 days CABG. Hospital average stay of 7.1 days TMLR + CABG vs. 8.2 days CABG. 30-day readmission rate 2.8% TMLR + CABG vs. 7.8% CABG. MACE outcomes were similar in both groups. Postoperative AFIB rates 16.7% TMLR + CABG vs. 37.4% CABG
Dziuk et al. (2003) [26]	62 patients: TMLR + CABG (n = 24) and TMLR only (n = 38)	6 months	Angina score by Canadian Cardiovascular Society, exercise tolerance, left ventricular function	Similar improved exercise tolerance and angina reduction in both groups. LVEF decreased from 61% to 54% at six months post-operation in both groups
Lutter et al. (2000) [27]	97 patients: CABG only (n = 35), TMLR only (n = 45), TMLR + CABG (n = 17)	12 months	Angina score by Canadian Cardiovascular Society, NYHA class, mortality, exercise tolerance, echocardiography with radionuclide assessments of metabolism and perfusion	Significantly decreased angina severity and NYHA class reduction in all groups. 0% mortality TMLR + CABG vs. 11% TMLR vs. 11% CABG. Treadmill exercise tolerance significantly improved in all groups. Stress-induced perfusion increase in CABG vs. no changes TMLR or TMLR + CABG
Konstanty-Kalandyk et al. (2018) [28]	86 patients: TMLR + CABG (n = 46), single therapy of either CABG or TMLR (n = 40)	10 years	10-year survival rate, Kaplan-Meier analysis, stratified by location on the heart wall	10-year survival 78.3% TMLR + CABG vs. 72.5% in single therapy. Stratified 10-year survival rate 100% TMLR + CABG vs. 72% single therapy with TMLR on anterior wall. TMLR revascularization showed no statistical differences between TMLR + CABG vs single therapy on posterior or lateral walls
Guleserian et al. (2003) [29]	81 patients: TMLR + CABG (n = 47) and TMLR alone (n = 34)	24 months	Mortality assessment	No significant difference in overall mortality and stratified mortality. Mortality decreased in the stratified category with patients who had an ejection fraction <40 with 62% mortality at 18-month follow-up compared to 90% in patients who did not have an ejection fraction <40. Stratified mortality decreased to 48% in CHF patients at 18 months vs. 96% in non-CHF

Discussion

The addition of TMLR to CABG has been a topic of ongoing research and debate in the surgical treatment of patients with CAD. Multiple studies have examined the short-term and long-term outcomes of this combined approach compared to either procedure performed alone. In the existing literature, the safety and feasibility of the combined TMLR and CABG procedure have been generally established, with short-term outcomes superior to those observed with TMLR or CABG alone. However, the long-term benefits appear to be more nuanced and dependent on specific factors. In this discussion, the key findings from the literature are summarized, i.e., safety and efficacy of the combined procedure, short-term and long-term outcomes, especially as related to the location of TMLR channels, and quality of life considerations.

The existing literature has generally established the safety and feasibility of the combined TMLR and CABG procedure, with short-term outcomes showing superiority over either TMLR or CABG alone. However, the long-term benefits appear to be more nuanced and dependent on specific factors, such as the location of TMLR channels. Studies have demonstrated a clear survival benefit when the TMLR component is performed on the anterior heart wall compared to TMLR on other walls or CABG alone. Additionally, the combined TMLR and CABG approach has been shown to provide improved quality of life and angina relief, particularly for high-risk patients. However, caution should be exercised when considering this approach for patients with left ventricular dysfunction or heart failure, as their long-term survival may be decreased.

*Safety and Efficacy of the Combined TMLR and CABG* *Approach*

The existing body of literature has generally established the safety and feasibility of the combined TMLR and CABG procedure as a treatment option for patients with CAD. In the short-term one-year studies published by Trehan et al. in 1997 and 1998 [17,18], the combined TMLR and CABG approach had an operative mortality of 1.3%-1.8% due to ventricular arrhythmia in the two studies from a combined total of 133 patients across the two studies. This mortality rate appears favorable when compared to mortality rates reported for CABG alone in several large registries of coronary surgery, although more research is needed to directly compare the two approaches. In addition, patients who underwent the combined TMLR and CABG procedure demonstrated a higher rate of freedom from MACE at 92% compared to 86% in the CABG-only group in the Allen et al. study [20,21].

Similarly, in the larger study published by Berishvili et al. [23] in 2015, including 831 patients, operative mortality was significantly decreased in patients with diffuse CAD who received the combined TMLR and CABG approach compared to CABG alone. The existing literature suggests that the combined TMLR and CABG procedure can be performed safely, with low short-term mortality and morbidity rates that are comparable or even superior to CABG alone in patients with diffuse coronary lesions not amenable to traditional revascularization. However, caution must be exercised when considering this approach for patients predisposed or susceptible to ventricular arrhythmias.

The existing evidence suggests that the combined TMLR and CABG procedure represents a reasonable treatment option for selected patients with diffuse CAD after carefully weighing the risks and benefits. However, the need for external validation through well-designed comparative effectiveness research remains dependent on utilizing clinical trials with larger sample sizes to further establish the safety profile and long-term outcomes of this combined approach. While the short-term outcomes have shown promise, longer-term data is still needed to fully understand the comparative benefits and identify the patient populations most likely to derive enduring clinical benefits from this combined surgical intervention. Rigorous, multi-center clinical studies with adequate statistical power and extended follow-up durations will be crucial to providing high-quality evidence to guide clinicians in the appropriate selection and management of patients with complex CAD.

Short-Term Outcomes and Benefits of the Combined TMLR and CABG Approach

Looking at short-term outcomes, multiple studies have demonstrated superior outcomes with the combined TMLR and CABG approach compared to either procedure alone. For example, patients who underwent the combined TMLR and CABG procedure in the study by Allen et al. in 2000 [19] had a one-year survival rate of 95% compared to 89% in the CABG-only group. Additionally, a follow-up study five years later showed that the combined TMLR-CABG group had lower mean angina scores, a lower proportion of patients with class 3/4 angina, and a greater trend toward being angina-free compared to the CABG-alone group. These findings suggest that the combined TMLR and CABG approach may provide improved short-term outcomes and better symptom relief for patients with CAD. The Loubani et al. [24] study in 2003 indicated a similar trend, with the combined TMLR and CABG group showing significant improvements in exercise tolerance testing compared to the CABG-alone group; however, this beneficial effect was lost at 36 months. It is important to note that this study used a patient sample size of 20 patients, limiting the statistical power to detect differences. The Lutter et al. [27] study in 2000 examined several parameters, including angina, mortality, and cardiac assessment, in surgical cohorts receiving either combined TMLR and CABG therapy or single therapy with either CABG only or TMLR only. The study population totaled 97 patients. Notably, the combined TMLR and CABG group had a 0% mortality rate at 12 months, in contrast with an 11% mortality rate in both of the single therapy groups. However, the other parameters evaluated showed no statistically significant differences between the groups.

The study by Wehberg et al. [25] in 2003 examined patients receiving either CABG alone or the combined TMLR and CABG approach, analyzing outcomes over a 30-day period. The authors reported that patients who underwent the combined TMLR and CABG procedure experienced significantly lower incidences of postoperative atrial fibrillation, as well as significantly reduced rates of intensive care unit stay, 30-day hospital readmissions, and an overall shorter length of hospital stay compared to those who received CABG alone. These findings suggest that the combined TMLR and CABG approach may offer improved short-term outcomes and a more favorable postoperative recovery profile for select patients with CAD. While this study had a relatively large patient population of 255 individuals, the number of patients who received the combined TMLR and CABG procedure was relatively small, with only 36 patients in this group, which may affect the generalizability of the results. Similarly, the study performed by Dziuk et al. [26] in 2003 utilized a small patient population of 62 individuals, with only 24 receiving the combined TMLR and CABG procedure compared to 38 patients receiving TMLR only. Unlike the other studies mentioned so far, this study did not find any statistically significant differences in short-term six-month outcomes, such as a reduction in the severity of angina or improvement in exercise tolerance, between the combined TMLR and CABG group and the TMLR-only group; however, the left ventricular ejection fraction decreased in both patient cohorts postoperatively, indicating caution might be warranted in patients with compromised or potential compromised ventricular function. In a similar protocol, in 2003, Guleserian et al. [29] published the results of a study evaluating outcomes over 24 months in a patient population of 81 individuals, with 47 patients receiving combined TMLR and CABG and 34 patients receiving TMLR alone. The authors reported that the TMLR-only patient cohort experienced a diminished quality of life compared to the combined TMLR and CABG group. Furthermore, the authors stratified the patient groups by certain high-risk surgical features and found that all patients regardless of the type of surgical intervention received, who had a reduced ejection fraction of less than 40% or congestive heart failure, had a significantly increased mortality risk at 18 months compared to patients without these risk factors. Specifically, the authors found that patients with a reduced ejection fraction below 40% or congestive heart failure had a significantly higher mortality rate at 18 months compared to patients without these high-risk factors, regardless of whether they received the combined TMLR and CABG procedure or TMLR alone. This suggests that the presence of reduced ventricular function or heart failure may be a critical determinant of long-term outcomes, and clinicians should carefully consider these patient characteristics when deciding between the combined TMLR and CABG approach and TMLR alone.

In summary, the current body of literature suggests short-term benefits of the combined TMLR and CABG approach over single revascularization therapies, including improved survival, reduced angina, and better exercise capacity. While these findings are promising, the existing data has limited external validity due to small sample sizes and heterogeneous patient populations. Larger, well-powered clinical studies with longer follow-up durations are still needed to better understand the comparative effectiveness of the combined TMLR and CABG approach and to provide more definitive results that can be reliably applied to real-world clinical practice. Additional research is necessary to elucidate the specific factors that may influence the long-term outcomes, such as the location of the TMLR channels, and to identify the patient populations that are most likely to benefit from this combined surgical intervention.

Long-Term Outcomes and Benefits of the Combined TMLR and CABG Approach

While the studies mentioned so far have shown generally positive short-term results with the combined TMLR and CABG approach, the longer-term outcomes are more nuanced. Furthermore, higher-quality clinical studies with larger sample sizes and longer follow-up durations are still needed in this area to draw more definitive conclusions. The results of the Allen et al. [20] study published in 2004, which followed 218 patients for up to five years, provide some insights into the long-term effects of this combined surgical intervention. Over the five-year follow-up period, patients who underwent the combined TMLR and CABG procedure exhibited significantly improved angina symptoms. There was an overall decrease in the number of patients experiencing severe and refractory angina in this group, and they also displayed a greater trend toward being completely angina-free compared to those who received CABG alone. These findings suggest that the combined TMLR and CABG approach may provide superior long-term relief from debilitating chest pain and ischemic symptoms for select patients with advanced CAD. However, there was no significant difference in overall survival rates between the two groups over the five-year follow-up period. Similarly, in the study published by Frazier et al. [22] in 2004, which followed 44 patients for up to four years, there was no statistically significant difference in long-term mortality rates between the combined TMLR and CABG and CABG-only groups. However, the authors concluded that patients who received the combined TMLR and CABG procedure had significantly higher freedom from MACE and other adverse cardiovascular outcomes over the four-year follow-up period and had zero revascularization procedures compared to 24% of the CABG-only group who required repeat revascularization.

The study by Konstanty-Kalandyk et al. [28] in 2016 evaluated the long-term survival and clinical outcomes of 86 patients with advanced coronary disease and refractory angina. The researchers compared patients who underwent the combined TMLR and CABG procedure to those who received a single therapy of either TMLR or CABG alone. Additionally, the researchers performed Kaplan-Meier survival analysis to examine the impact of the location of the TMLR channels, whether they were placed on the anterior, posterior, or lateral ventricular wall, on long-term survival and outcomes. This analysis provided insights into the potential differential effects of TMLR channel placement on patient prognosis. The authors reported that patients who received the combined TMLR and CABG intervention exhibited higher long-term survival rates compared to those who underwent TMLR or CABG alone over the 10-year follow-up period, although this difference was not statistically significant. Furthermore, they found that patients who had TMLR channels placed on the anterior ventricular wall exhibited significantly higher 10-year survival rates compared to those with posterior or lateral wall channel placements. Specifically, the stratified 10-year survival rate was 100% in the TMLR + CABG group versus 72% in the single therapy group that had TMLR performed on the anterior heart wall. These findings highlight the potential importance of TMLR channel location as a critical factor influencing long-term clinical outcomes. The location of the TMLR channels, whether on the anterior, posterior, or lateral ventricular wall, may have a significant impact on patient prognosis and survival over the long term. This suggests that the careful selection and placement of TMLR channels could be an important consideration when determining the optimal treatment approach for patients with advanced coronary disease and refractory angina.

Despite the promising evidence from this study, the authors also reported that patients with complex, multivessel, disseminated coronary lesions had an overall increased cardiac mortality six years after the initial revascularization procedures, regardless of whether they received the combined TMLR and CABG approach or single therapies.

## Conclusions

Current evidence suggests the combined TMLR and CABG approach may provide improved short-term benefits. Long-term clinical outcomes and comparative effectiveness of combined surgical intervention are mixed. Some studies reported higher long-term survival rates with lower rates of adverse cardiovascular events with combined TMLR and CABG, while other studies found no significant differences in long-term mortality when compared to CABG only. Long-term prognosis with the combined approach may be affected by the location of the TMLR. Prognostic factors positively influencing postoperative outcomes and long-term survival may include TMLR channel placement on the anterior ventricular wall rather than the posterior or lateral walls. The reviewed evidence indicates patients with diffuse and complex coronary lesions have superior short-term and intraoperative outcomes compared to single therapy cohorts; however, this specific patient population maintains an elevated long-term cardiac mortality risk regardless of the revascularization modality used. Patients with congestive heart failure or reduced left ventricular function may derive less long-term benefit from the combined TMLR and CABG approach. One of the main limitations of the current evidence is the relatively small sample sizes and heterogeneity of the study populations across the available clinical trials. Additional large-scale, well-designed studies with longer follow-up periods are still needed to more definitively elucidate the precise long-term clinical impact, cost-effectiveness, and patient selection and risk stratification criteria for this combined surgical approach, especially compared to other standard revascularization techniques. The comparative long-term effectiveness of the combined TMLR and CABG approach versus single revascularization therapy remains an open question that could benefit from additional research.
